# The updated RGD Pathway Portal utilizes increased curation efficiency and provides expanded pathway information

**DOI:** 10.1186/1479-7364-7-4

**Published:** 2013-02-05

**Authors:** G Thomas Hayman, Pushkala Jayaraman, Victoria Petri, Marek Tutaj, Weisong Liu, Jeff De Pons, Melinda R Dwinell, Mary Shimoyama

**Affiliations:** 1 Rat Genome Database, Human and Molecular Genetics Center, Medical College of Wisconsin, Milwaukee, WI 53226, USA

**Keywords:** Curation, Databases, Ontologies, Pathways, Tools

## Abstract

The RGD Pathway Portal provides pathway annotations for rat, human and mouse genes and pathway diagrams and suites, all interconnected via the pathway ontology. Diagram pages present the diagram and description, with diagram objects linked to additional resources. A newly-developed dual-functionality web application composes the diagram page. Curators input the description, diagram, references and additional pathway objects. The application combines these with tables of rat, human and mouse pathway genes, including genetic information, analysis tool and reference links, and disease, phenotype and other pathway annotations to pathway genes. The application increases the information content of diagram pages while expediting publication.

## Overview

The laboratory rat (*Rattus norvegicus*) has been used as an animal model for over 150 years, with inbred strains used to study human physiology and many human diseases, such as autoimmune, cardiovascular, kidney, and pulmonary diseases, and metabolic, reproductive, and urogenital disorders [1]. The Rat Genome Database (RGD; http://rgd.mcw.edu) [2] is the primary archive of rat genetic and genomic data, holding over 40,000 active rat gene records, plus human and mouse orthologs. In addition to protein-coding genes, these include RNA genes and pseudogenes. A recent analysis reported 17,733 unambiguous rat orthologs of human genes, occurring in many syntenic segments [3], further supporting the use of the rat as a model for human physiology and disease. Also housed at RGD is information on rat and human quantitative trait loci (QTLs) and rat strains accumulated by manual curation [4] using an advanced suite of curation tools [5] and automated pipelines. RGD uses over a dozen different ontologies to annotate gene, QTL, and strain information. It is one of the few databases that stores human QTL data.

The Pathway Portal project [6] at RGD aims to provide a dynamic platform where users can find pathway associations for human, rat, and mouse genes. Users can access interactive pathway diagram pages, suites of functionally related pathways, and suite networks illustrating broader interactions to explore the connections between these and across other available resources. Several entry points and tools accessible from the RGD home page allow the user to search for and access pathway data and navigate between the portal’s components and the other resources at RGD. Searching for a pathway ontology (PW) term in the RGD generic keyword search will bring up a link to the pathway report page. The RGD Disease Portals also provide links to the pathway report pages. Pathway curation for the Pathway Portal involves annotating human, rat, and mouse genes to terms in the PW, which was originated at and continues to evolve at RGD. Curated pathway data are derived largely from published scientific review literature, and the norm is to annotate to the human genes; corresponding annotations made to the rat and mouse orthologs are qualified with the evidence code ‘inferred from sequence similarity’ (ISS) to denote that the annotation is additionally predicted for the rodent genes based on shared sequence similarity with the human gene. It might be suggested that use of pathway terms in the biological process arm of the Gene Ontology (GO) would be adequate for pathway annotations. While there are overlapping terms between PW and GO, the latter uses the perspective of a unidirectional process whose reactions and interactions lead to an end result, while the PW perspective is one of sets of interacting molecules whose reactions and interactions underlie functioning networks. This pathway ontology has five major nodes: metabolic, regulatory, signaling, drug, and disease pathways, with terms for altered versions of pathways. Drug pathways are offered by PharmGKB (http://www.pharmgkb.org/) and the Small Molecule Pathway Database (http://www.smpdb.ca/) with the latter also having disease along with signaling and metabolic pathways. Disease pathways can be found at the Kyoto Encyclopedia of Genes and Genomes (KEGG; http://www.genome.jp/kegg/) and Reactome (http://www.reactome.org/ReactomeGWT/entrypoint.html). RGD’s PW is unique in having all pathway types including disease and altered (unique to PW) versions. The ontology allows for the standardized annotation of genes to pathway terms, provides a link to interactive pathway diagrams, and serves as a navigational tool between the various pathway data types. The generation of diagrams and interactive diagram pages are important components of the curation process for the Pathway Portal. The visualization of pathways aids the user in understanding the position of and relationships between gene functions within networks. An application has been developed both to increase the range of and accessibility to the biological information provided by pathway diagram pages, and to expedite their production.

## The Molecular Pathway diagram pages web application

**Figure 1:**
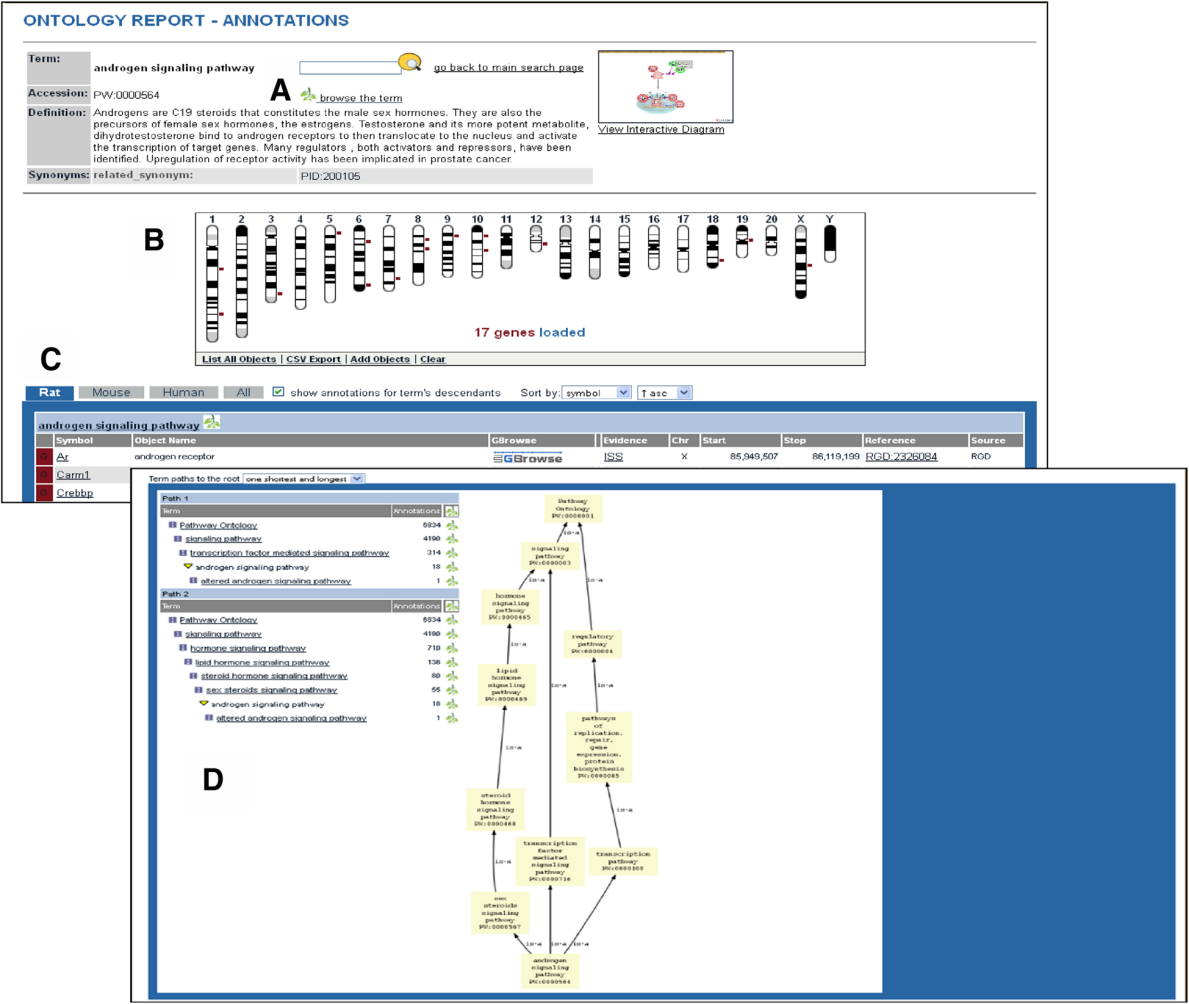
**Updated ontology report.** (**A**) Term definition, browser link, and diagram icon. (**B**) Genome Viewer display. (**C**) Pathway gene member table. (**D**) Choice of ontology term path with annotation counts.

**Figure 2:**
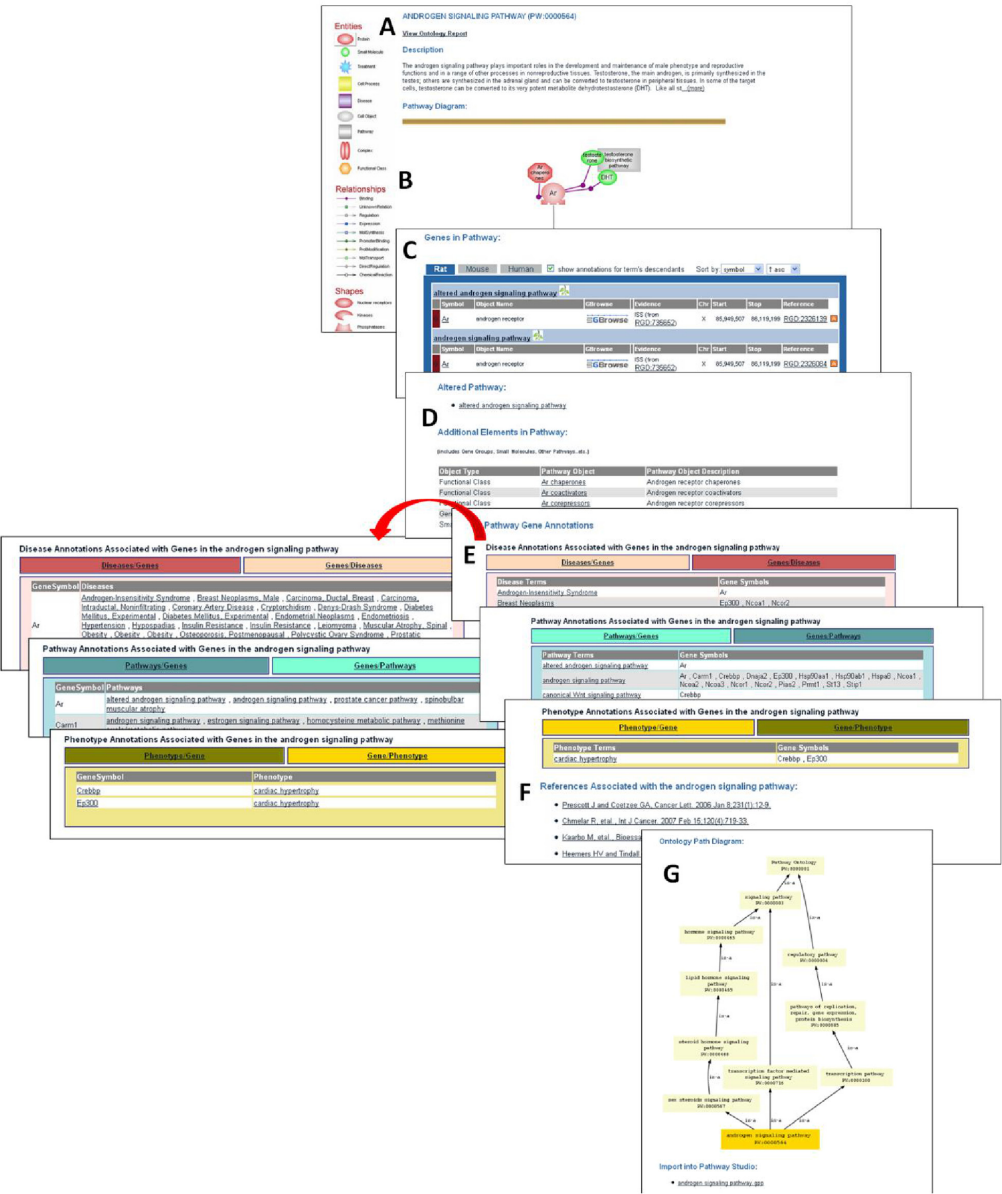
**Updated pathway page.** (**A**) Expandable description. (**B**) Pathway diagram. (**C**) Pathway gene member table. (**D**) Additional pathway elements table. (**E**) Tables of disease, other pathway, and phenotype terms annotated to pathway member genes. (**F**) References annotated to the pathway. (**G**) Ontology path diagram.

A new molecular pathway application has been developed for the Pathway Portal that has two functions. First, it greatly expands the amount of pathway information available to the user. The diagram pages generated using the application are copied to the public database, where they can be accessed by users from the Pathway entry points of the RGD home page. Pathway terms can be searched using the ontology browser, the Genome Viewer tool, keyword search or entries in the Disease Portals, all found on the RGD home page. The result is a recently revised ontology report (Figure 1) with a definition and link to the improved ontology browser (Figure 1A) [7]. A genome-wide Genome Viewer map shows the chromosomal positions of genes annotated to the pathway (Figure 1B). The list of these pathway member genes, with chromosome locations, is downloadable via the comma-separated variable export link at the bottom of the Genome Viewer, using spreadsheet software. The RGD database is parsed to generate a table, which is tabbed to select lists of rat, human, or mouse genes annotated to the term and, if selected, term children. Additional genetic and annotation information is included, along with a link to the RGD GBrowse tool, which now has human, rat, and mouse versions for further sequence analysis (Figure 1C). The reader is referred to Laulederkind et al. [8] for a detailed description of the use of GBrowse and other RGD analysis tools. The table is sortable by several parameters. A choice of views of ontology tree paths for the term is presented, including the number of existing annotations at each level (Figure 1D). If available, the icon of an interactive pathway diagram is displayed (Figure 1A) which links to the recently enhanced diagram page (Figure 2). The pathway diagram page contains an expandable description (Figure 2A) that includes curator-established links to Pfam entries for domains that are mentioned in the pathway description, such as phosphotyrosine binding and Src homology domains mentioned in the insulin signaling pathway report page description (not shown). More recently, links have been provided to Research Collaboratory for Structural Bioinformatics Protein Data Bank entries for structures, as in the visual phototransduction pathway report page, and links to KEGG, Reactome, and GO term entries as applicable. The diagram itself contains links to gene report pages, provided from gene icons depicted in the pathway (Figure 2B) as well as from the same Genes in Pathway table of member genes presented in the ontology report (Figure 1C and Figure 2C). Additional new lists of other pathway elements such as members of a gene family or target genes (individual entries link to gene report pages), as well as descriptions and PubChem or Chemical Entities of Biological Interest (ChEBI) links for small molecules are supplied (Figure 2D; the molecule icons in the diagram also link to this information). Tables that are automatically compiled by the web application provide parsed information about diseases, other pathways, and phenotypes annotated to pathway gene members. These can be toggled between listing alphabetically by gene or by disease, pathway or phenotype (Figure 2E). If the user is interested in one or several genes, these tables offer immediate information on their disease, phenotype, and other pathway associations without the need to go to the individual report pages. This information is available for all genes in the pathway. A list of references annotated to the pathway term (Figure 2F) and an ontology path diagram are also furnished, along with a link to download the diagram for users of the Ariadne software (Figure 2G). A version of the diagram can be saved by right-clicking on the diagram background, then saving the image as a portable networks graphics file. The ability to download these gene lists and associations, and the diagram using freely available software is in development. Importantly, the Ariadne software includes the expandable ResNet mammalian database, which contains entries for human, rat, and mouse genes, small molecules, diseases, and processes with accessory data. The fact that new features can be added to the database has been exploited to generate informational links to small molecules described above and links to pathways triggered by or connected to the pathway under investigation (Figure 2B, testosterone biosynthetic pathway), or between a disease pathway and the underlying altered pathways (Figure 2C, D, altered androgen signaling pathway). This feature allows users to ‘travel’ through the pathway landscape, comparing normal to altered pathways to examine the potential role alterations may play in disease. 

**Figure 3:**
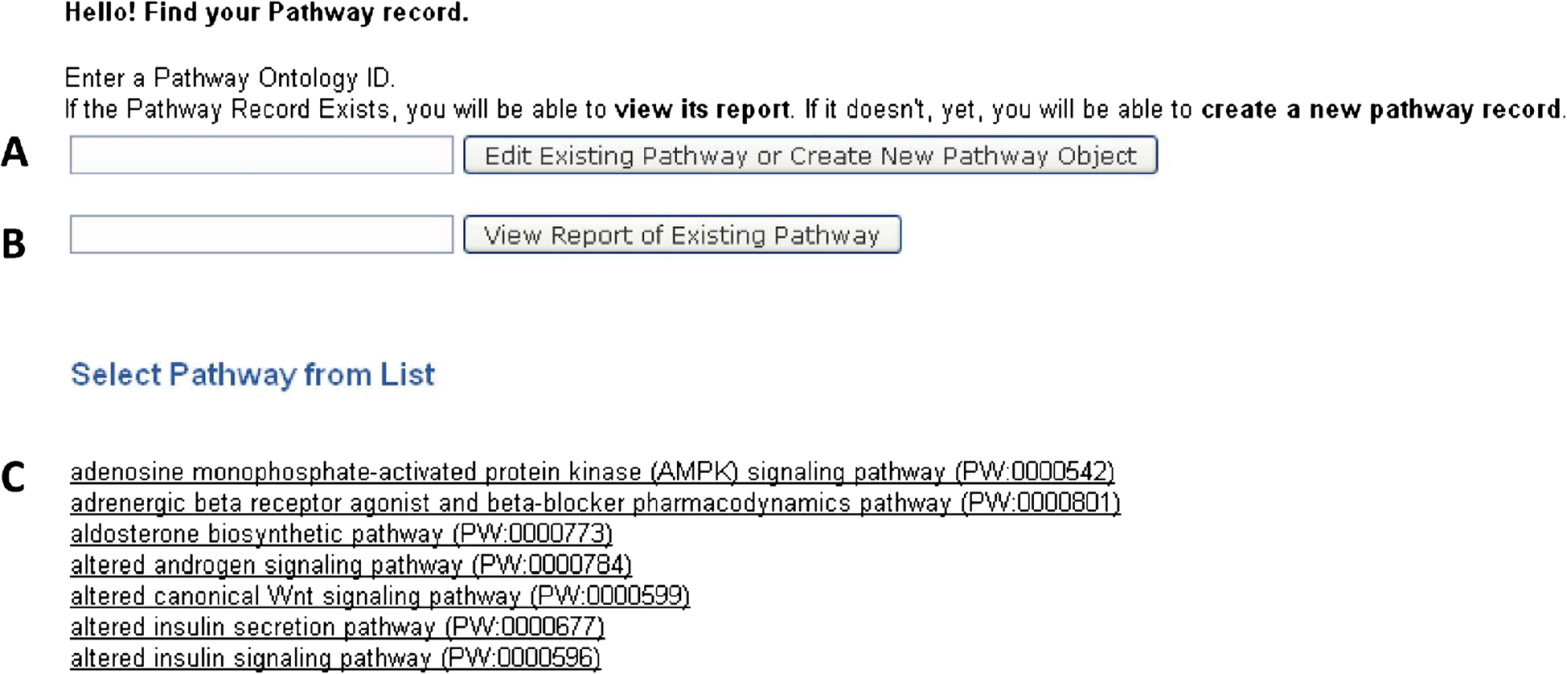
**Pathway application Creation/Edit entry page.** (**A**) A pathway ID is entered to generate a new page or edit an existing one. (**B**) Existing pages can be viewed by entering the pathway ID. (**C**) List of existing pathway report pages.

**Figure 4:**
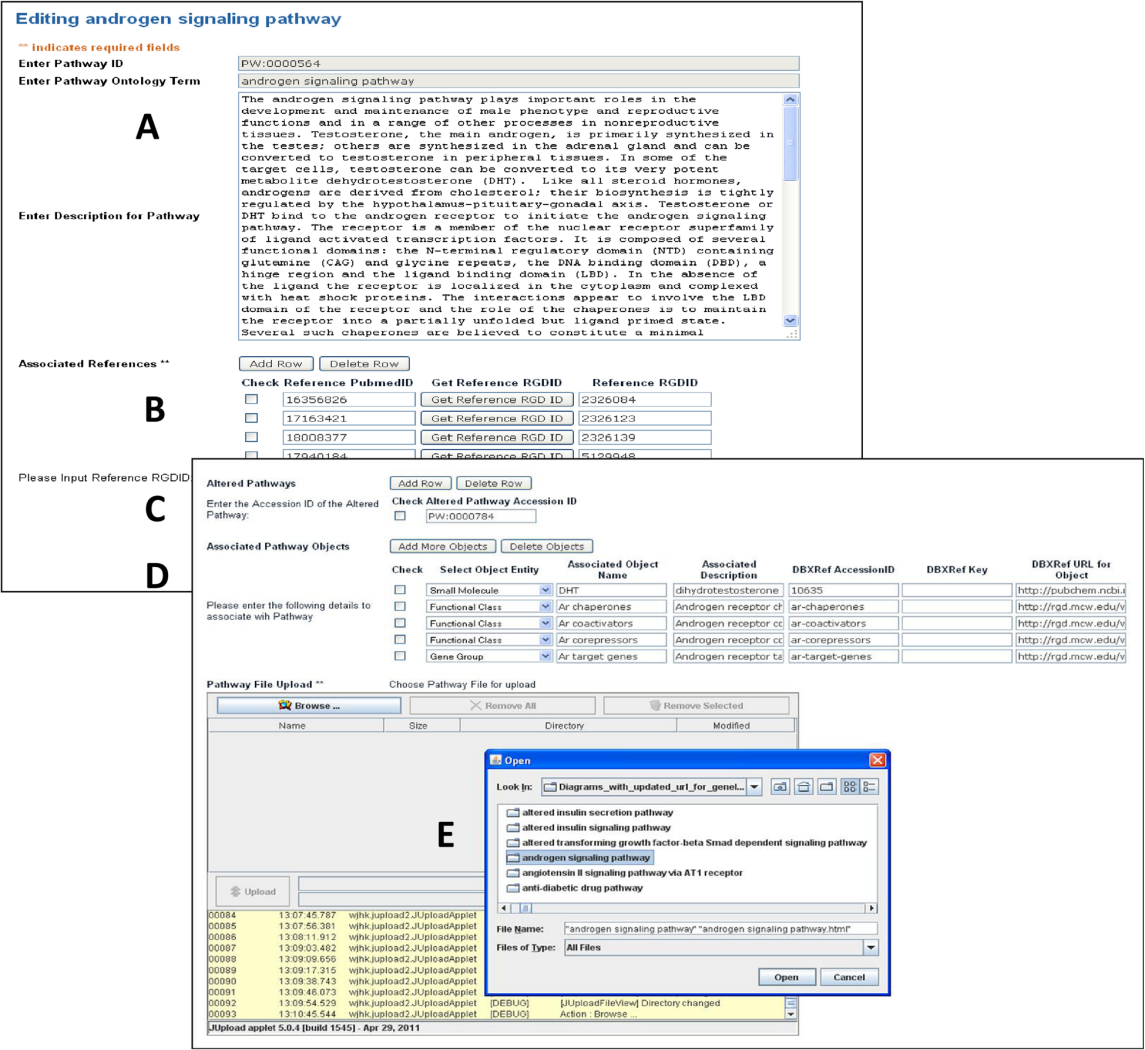
**Creation/Edit interface.** (**A**) Description entry/editing. (**B**) Reference entry and conversion. (**C**) Altered pathway entry. (**D**) Associated pathway objects entry. (**E**) Diagram upload.

The second function of the pathway application is to greatly streamline the establishment by curators of the many relationships displayed on the pathway pages. Previously, this was a complex, detailed, involved, and slower process. The streamlining process using the new application consists of two components. The first component is a template for creating a pathway report page. This includes a description, a list of associated references, linked pathway objects, and altered pathways, all of which are editable. The template also uploads the pathway diagram. The Pathway Creation/Edit entry page (Figure 3) allows curators to search for pathways either via PW term accession numbers or by entering the term name (Figure 3A). An autocomplete function facilitates this process. Also listed are all the pathways that have already been created and stored in the database for ease of access (Figure 3C). Searching for a pathway that is not present in the database will automatically redirect the page to an interface form (see Figure 4), allowing a curator to create the pathway diagram page. The entry page contains pre-stored pathway Term and Accession information extracted from the database. Searching for an existing pathway using the first text box (Figure 3A) opens the editing interface for that pathway. A search using the second text box (Figure 3B) results in a view of the pathway page (see Figure 2). The Creation/Edit interface consists of five sections (Figure 4). The description is entered or edited in the first text window, and links to other databases within the text are established by the curator (Figure 4A). In the second section, references used for pathway annotations are added, either as PubMed IDs, which are automatically converted into RGD IDs, or as RGD IDs directly (Figure 4B). Term IDs for any altered pathways are entered in the third section (Figure 4C). Additional pathway element identities, descriptions, and PubChem or ChEBI links are supplied in the fourth section (Figure 4D). In the references, altered pathways, and additional pathway elements sections, new items can be created or existing ones deleted. Lastly, the requisite interactive diagram files and folders, generated using Ariadne Pathway Studio software (version 8.0), are uploaded for display (Figure 4E). The links established within the diagram as it was generated using the Ariadne software are retained by the pathway application. When the information is updated at the bottom of the interface, the curator is taken to the pathway report page (see Figure 2) to confirm that all entered information is displayed. In the second component of the streamlining process, the application automatically parses the database to produce the informational tables described above, which display disease, phenotype, and other pathway annotations made to the genes in the pathway being studied. A view of the ontology path for the PW term is also added. 

## Software development

The Molecular Pathway application is built on J2EE (http://java.sun.com/j2ee/overview.html) technologies and driven off the RGD Oracle database. It can be run on any Java container that implements the Servlet 2.4 and JavaServer Pages 2.0 specification or above. The web application is built on the Spring [9] framework’s model-view controller architecture. In order to make ontology term entry more efficient, an ontology term autocomplete feature was implemented based on Apache Solr (http://lucene.apache.org/solr/) and JQuery (http://jquery.com/). The feature can also convert ontology terms to ontology IDs. The application utilizes asynchronous Javascript and XML (AJAX) [10] allowing the curator to add and delete fields without a refresh of the page. In addition, an AJAX quick retrieve has been included to allow for quick translation from PubMed reference ID to RGD ID without the need for a new page. A common gateway interface (CGI) program that handles abstract download and RGD ID assignment has been modified so that the CGI program can accept the PubMed ID from the pathway editing interface and automatically send the result back to the same interface. This program not only returns an RGD ID for an already existing reference but also downloads the reference abstract, assigns a new RGD ID, and returns the newly created RGD ID back to the interface in the case of a reference not present in the database.

Pathway Image files created in the Ariadne Pathway Studio software tool (version 8.0) are uploaded with Jupload, an OpenSource applet distributed on SourceForge that allows multiple file uploads within a single directory. This applet is distributed under the Common Public License version 1.0: JUnit, GNU GPL (general public license) V3: JUpload project on SourceForge, and The Apache Software License version 2.0: Commons Lang, Jakarta Commons Net, Maven Plugin application programming interface (http://jupload.sourceforge.net/). The compiled JUpload applet, signed with the JUpload project certificate, is employed for upload of the pathway directories to the curation server. A Java class that inherits the Hypertext Markup Language (HTML) Parser and Node Visitor methods is used to replace links created by Pathway Studio with links to all of the RGD resources. Automatic regeneration of file structure ensures no manual intervention during upload of each new or updated pathway.

New objects and updates to existing objects are run through a validation layer to reduce the probability that errors make it into the database. Uploaded files are automatically scanned to ensure the correct pathway files are loaded. Supported browsers include IE 8+, Firefox 3+, and Safari 5+. In addition, built-in validation routines are included in the page to assist curators in quality control. The user interface is built on standard web technologies including HTML, JavaScript, and cascading style sheets. After creating or editing a pathway, a curator is given an ‘almost-final’ view of the created pathway which can be further edited.

The architecture and capabilities of the Pathway Curation software may be of interest to the informatics community. However, because the pathway application software was designed specifically to fit the RGD database schema and uses the licensed Ariadne software, it would not be readily adaptable for use with other databases, so it has not been made publicly available.

## Perspective and outlook

The RGD Pathway Portal continues to grow, as does the number of researchers using it. A data pipeline importing almost 24,000 pathway gene annotations, including over 8,000 for human genes, from the Pathway Interaction Database (http://pid.nci.nih.gov/) has been recently added. A similar pipeline importing pathway data from KEGG will be released shortly. There are presently over 9,400 pathway annotations and in excess of 100 interactive pathway pages published at RGD. These are of great interest to research community users as evidenced by the numbers of views the pages receive (43,333 total, 27,547 unique page views from August 01, 2011 to August 01, 2012), determined using Google Analytics, as well as by upgrades and new trends found at other data sources. These include new webinars introducing the pathway and network visualization and analysis tools at Reactome, and the new Reaction Modules at KEGG.

In the process of providing the dynamic platform or ‘landscape’ for exploration that the Pathway Portal strives to be, new approaches or views are developed, such as the pathway suites and suite networks. Each suite offers an instant snapshot of the broader picture that brings together several pathways. The suite networks interconnect related pathway suites, illustrating their complex, higher-order interactions. We look forward to continued growth in all of these areas. As the portal expands in depth and scope, other approaches and tools will be developed and added to enhance the pathway page collection at RGD and the value it offers to the research community.

## Abbreviations

AJAX: asynchronous Javascript and XML; CGI: common gateway interface; ChEBI: Chemical Entities of Biological Interest; GO: Gene Ontology; HTML: hypertext markup language; KEGG: Kyoto Encyclopedia of Genes and Genomes; PW: pathway ontology; RGD: Rat Genome Database.

## Competing interests

The authors declare that they have no competing interests.

## Authors’ contributions

GTH wrote the manuscript and evaluated the software tool and report pages. PJ designed the application, redesigned the report pages, and assisted in writing the manuscript. VP evaluated the application and report pages, and assisted in writing the manuscript. MT, WL, and JDP participated in software development and report page redesign. MRD is the co-principal investigator. MS is the principal investigator and participated in the design and coordination of the application. All authors read and approved the final manuscript.

## References

[B1] Jacob HJ Functional genomics and rat models Genome Res 1999 9 1013 1016 10.1101/gr.9.11.1013 10568741

[B2] Shimoyama M Smith JR Hayman T Laulederkind S Lowry T Nigam R Petri V Wang SJ Dwinell M Jacob H Team RGD RGD: a comparative genomics platform Hum Genomics 2011 5 124 129 21296746 10.1186/1479-7364-5-2-124 PMC3222152

[B3] Kemkemer C Kohn M Cooper DN Froenicke L Högel J Hameister H Kehrer-Sawatzki H Gene synteny comparisons between different vertebrates provide new insights into breakage and fusion events during mammalian karyotype evolution BMC Evol Biol 2009 9 84 10.1186/1471-2148-9-84 19393055 PMC2681463

[B4] Shimoyama M Hayman GT Laulederkind SJ Nigam R Lowry TF Petri V Smith JR Wang SJ Munzenmaier DH Dwinell MR Twigger SN Jacob HJ Team RGD The rat genome database curators: who, what, where, why PLoS Comput Biol 2009 5 e1000582 10.1371/journal.pcbi.1000582 19956751 PMC2775909

[B5] Laulederkind SJF Shimoyama M Hayman GT Lowry TF Nigam R Petri V Smith JR Wang SJ De Pons J Kowalski G Liu W Rood W Munzenmaier DH Dwinell MR Twigger SN Jacob HJ Team RGD The Rat Genome Database curation tool suite: a set of optimized software tools enabling efficient acquisition, organization, and presentation of biological data Database 2011 2011 bar002 10.1093/database/bar002 21321022 PMC3041158

[B6] Petri V Shimoyama M Hayman GT Smith JR Tutaj M De Pons J Dwinell MR Munzenmaier DH Twigger SN Jacob HJ Team RGD The Rat Genome Database pathway portal Database 2011 2011 bar010 10.1093/database/bar010 21478484 PMC3072770

[B7] Laulederkind SJ Tutaj M Shimoyama M Hayman GT Lowry TF Nigam R Petri V Smith JR Wang SJ De Pons J Dwinell MR Jacob HJ Ontology searching and browsing at the Rat Genome Database Database 2012 2012 bas016 10.1093/database/bas016 22434847 PMC3308169

[B8] Laulederkind SJF Hayman GT Wang SJ Lowry TF Nigam R Petri V Smith JR Shimoyama M Dwinell MR Jacob HJ Exploring genetic, genomic, and phenotypic data at the rat genome database Curr Protocols Bioinfin press 10.1002/0471250953.bi0114s40 PMC3555435 23255149

[B9] Walls C Breidenbach R Spring in Action 2007 2 Greenwich: Manning Publications

[B10] Crane D Pascarello E James D AJAX in Action 2005 Greenwich: Manning Publications

